# A cross-cohort analysis of autosomal DNA methylation sex differences in the term placenta

**DOI:** 10.1186/s13293-021-00381-4

**Published:** 2021-05-27

**Authors:** Amy M. Inkster, Victor Yuan, Chaini Konwar, Allison M. Matthews, Carolyn J. Brown, Wendy P. Robinson

**Affiliations:** 1 BC Children’s Hospital Research Institute, 950 W 28th Ave, Vancouver, V6H 3N1 Canada; 2 Department of Medical Genetics, University of British Columbia, 4500 Oak St, Vancouver, V6H 3N1 Canada; 3 Centre for Molecular Medicine and Therapeutics, 950 W 28th Ave, Vancouver, V6H 3N1 Canada; 4 Department of Pathology & Laboratory Medicine, University of British Columbia, 2211 Wesbrook Mall, Vancouver, V6T 1Z7 Canada

**Keywords:** DNA methylation, Placenta, Sex as a biological variable, Sex differences, Microarray, Illumina 450K array, Epigenetics, Pregnancy

## Abstract

**Background:**

Human placental DNA methylation (DNAme) data is a valuable resource for studying sex differences during gestation, as DNAme profiles after delivery reflect the cumulative effects of gene expression patterns and exposures across gestation. Here, we present an analysis of sex differences in autosomal DNAme in the uncomplicated term placenta (*n* = 343) using the Illumina 450K array.

**Results:**

At a false discovery rate < 0.05 and a mean sex difference in DNAme beta value of > 0.10, we identified 162 autosomal CpG sites that were differentially methylated by sex and replicated in an independent cohort of samples (*n* = 293). Several of these differentially methylated CpG sites were part of larger correlated regions of sex differential DNAme. Although global DNAme levels did not differ by sex, the majority of significantly differentially methylated CpGs were more highly methylated in male placentae, the opposite of what is seen in differential methylation analyses of somatic tissues. Patterns of autosomal DNAme at these 162 CpGs were significantly associated with maternal age (in males) and newborn birthweight standard deviation (in females).

**Conclusions:**

Our results provide a comprehensive analysis of sex differences in autosomal DNAme in the term human placenta. We report a list of high-confidence autosomal sex-associated differentially methylated CpGs and identify several key features of these loci that suggest their relevance to sex differences observed in normative and complicated pregnancies.

**Supplementary Information:**

The online version contains supplementary material available at 10.1186/s13293-021-00381-4.

## Background

Sex is a key variable influencing biological systems from the level of the cell to the level of the organism. Biological sex is typically defined by sex chromosome complement, which largely corresponds with the gonadal sex of the organism [1]. Biological sex is of particular importance in the study of human pregnancy and prenatal development as male fetal sex is a risk factor for several pregnancy complications including preterm birth, intrauterine growth restriction, and maternal gestational diabetes [2–6]. Sex differences during prenatal development are likely affected by sex differences in the placenta, the organ critical for regulating growth and development of the embryo/fetus throughout gestation. Except in rare cases, placental cells harbor the same sex chromosome complement as the fetus, and sex differences in placental function, for example placental response to infection and stress, could contribute to sex differences in fetal growth and development [5, 7, 8]. Placental DNA methylation (DNAme) data are a valuable resource for studying sex differences during gestation, as DNAme profiles after delivery reflect the cumulative effects of gene expression patterns and exposures across gestation.

In any tissue, when evaluating sex-specific DNAme both autosomal and X chromosomal loci should be considered. Sex differences in X chromosome DNAme patterns are extensive and expected, as DNAme plays a key role in the process of X-chromosome inactivation (XCI), by which one of the X chromosomes in female cells is epigenetically silenced [9, 10]. In contrast, the extent to which autosomal DNAme varies by sex is less clear. Initial reports of sex-specific autosomal DNAme were later deemed false positives, attributed to microarray probes with high sequence affinity to multiple genomic regions including X- or Y-linked loci [11, 12]. It is now common to exclude CpG sites measured by such probes prior to analysis of DNAme data, but rarely are sex differences at the remaining autosomal CpGs investigated. As a result, literature investigating sex differences in placental autosomal DNAme and gene expression patterns is sparse. However, the handful of studies conducted on placentae from uncomplicated pregnancies suggest that the placenta harbors an appreciable number of autosomal loci with sex-specific DNAme profiles [13, 14] and that up to 60% of sex-differentially expressed placental genes are autosomal [15, 16].

Epigenome-wide association studies have been conducted to investigate the effects of disease and exposures in pregnancy, generally focusing on autosomal variation. Disease-related EWAS of the placenta include preeclampsia [17–21] (reviewed in [22]), acute chorioamnionitis [23], intrauterine growth restriction [20, 24], and fetal birthweight [25], among others. Recent placental EWAS of environmental exposures and maternal phenotypes include investigations into heavy metals [26, 27], pollution [28, 29], maternal smoking [30], maternal stress [31], blood pressure [32], diabetes [33], body mass index, gestational weight gain, and dyslipidemia [34, 35]. Understanding how biological sex is associated with autosomal DNAme is an underexplored facet of prenatal epigenetic research, and may shed light on the factors contributing to sex differences observed in growth and development throughout gestation. This study seeks to comprehensively characterize sex differences in the uncomplicated, full-term (> 37 weeks of gestation) placental DNA methylome, with the aim of establishing a baseline of sex differences observed in the uncomplicated placenta.

## Methods

### Datasets

The discovery cohort was compiled from public placental Illumina Infinium HumanMethylation450 (450K) datasets including GSE73375 (*n* = 9, NC, USA) [36], GSE75428 (*n* = 289, Rhode Island Child Health Study, RI, USA) [37], GSE98224 (*n* = 9, Toronto, Canada) [38], GSE74738, GSE100197, GSE108567, and GSE128827 (*n* = 34, all Epigenetics in Pregnancy Complications Cohort, Vancouver, Canada) [20, 39–41]. These compiled data were used as described in Yuan et al. to generate PlaNET, the Placental DNAme Elastic Net Ethnicity Tool, for estimating genetic ancestry from placental DNAme data [40]. An independent North American dataset was used for replication, GSE71678 (*n* = 293, New Hampshire Birth Cohort Study, NH, USA) (Table 1).

**Table 1:** Demographic characteristics of discovery and replication cohorts

	Discovery			Replication		
	Female (*n* = 177)	Male (*n* = 164)	*p* value^*^	Female (*n* = 137)	Male (*n* = 156)	*p* value^*^
**Gestational Age**						
Weeks (mean (SD))	39.0 (± 1.1)	39.1 (± 0.9)	0.53	39.6 (± 1.1)	39.7 (± 1.0)	0.28
**Condition**						
Healthy term	100	100	0.02	114	125	0.10
SGA	37	45		9	4	
LGA	40	19		13	24	
**PlaNET Ancestry**^**§**^						
Coordinate 1 (mean (SD))	0.10 (± 0.25)	0.07 (± 0.23)	0.27	0.0009 (± 0. 0016)	0.0012 (± 0.0018)	0.000024
Coordinate 2 (mean (SD))	0.11 (± 0.26)	0.05 (± 0.14)	0.37	0.0038 (± 0.0270)	0.0032 (± 0.0103)	0.011
Coordinate 3 (mean (SD))	0.78 (± 0.36)	0.88 (± 0.27)	0.23	0.9951 (± 0.0279)	0.9956 (± 0.0111)	0.001

SD refers to standard deviation; SGA and LGA refer to small (< 10th centile) and large (> 90th centile) birthweight for gestational age within each sex, as assigned by the original publications

^*^p values represent male-female comparisons, from Wilcoxon rank-sum tests for continuous and Fisher’s exact test for categorical variables

^§^PlaNET outputs of DNAme-based ethnicity/ancestry probability values range from 0 to 1 and sum to 1 for each sample. Coordinate 1 is associated with probability of African ancestry, coordinate 2 with East Asian ancestry, and coordinate 3 with European ancestry [40]

### Verification of sample sex and identity

In both the discovery and replication cohorts, sample sex was verified by hierarchical clustering on β values from CpGs mapping to the X and Y chromosomes (*n* = 11,648), and on β values from 5 CpGs in the X inactivation center methylated proportionally to the number of chromosomes silenced by XCI [10]. Two major sample clusters were observed in each step, corresponding to XX and XY chromosome complements. Samples were confirmed to be male or female if both sex clustering checks agreed with the annotated sex.

Samples were evaluated for genetic uniqueness using functions from the ewastools R package [42]. Two apparent genetic duplicates were discovered in the replication cohort; both were excluded from downstream analyses. Following sex and identity verification, the rs probes on the 450K array (*n* = 65) and CpGs mapping to the X or Y chromosome (*n* = 11,648) were removed from the discovery and replication datasets.

### Data processing and ancestry estimation

The discovery cohort was subjected to probe filtering and normalization as described in Yuan et al*.* [40], the replication cohort was processed similarly and independently. Preterm samples (< 37 weeks’ gestation) and those affected by preeclampsia were excluded from both datasets. Briefly, CpGs removed were those targeted by non-specific probes [43, 44], placental non-variable CpGs (range of β values < 0.05 between the 10th and 90th centile in all samples in these cohorts) [45], poor quality probes (detection *P* value > 0.01 or bead count < 3 in more than 1% of samples) [46]**,** and probes targeting polymorphic loci [43, 44]. The discovery cohort was normal exponential out-of-band (noob) and beta mixture quantile (BMIQ) normalized with functions from the wateRmelon and minfi packages [47]. The replication cohort was functional and noob normalized to correspond with the original publication of that dataset [26]. Using the PlaNET R package [40], samples were assigned three DNAme-estimated probabilities of arising from populations of African/Black, East Asian, and European/white descent, which sum to 1 in each sample. While PlaNET is a placental DNAme classifier trained on self-reported ethnicity, the output probabilities are significantly associated with both self-reported ethnicity and genetic ancestry and as such are typically referred to as coordinates [40]. In contrast to principal components analysis or multidimensional scaling-based methods of ancestry deconvolution, genetic ancestry variation is captured by adjusting for any two of the three PlaNET coordinates in statistical models [40]. This is similar to methods recommended for cell type adjustment in which any one of a set of compositional estimates is excluded from models to avoid overfitting [48]. After processing, the discovery cohort consisted of 324,104 autosomal CpGs in 341 samples suitable for sex-specific DNAme analysis, while 341,939 autosomal CpGs in 293 samples remained for replication analyses.

### Global sex-specific DNAme profile analyses

Sex differences in mean DNAme β values were tested at 324,104 filtered autosomal loci and 12,329 additional CpGs annotated to autosomal Alu and LINE1 repetitive elements by non-parametric Kruskal-Wallis tests. CpGs in repetitive regions were pulled from the non-probe-filtered dataset (*n* = 473,929 CpGs) by the overlap of Illumina probe locations and the UCSC hg19 RepeatMasker track [49]. Sex differences in cell type proportions (trophoblast, syncytiotrophoblast, stromal, endothelial, Hofbauer, and nucleated red blood cells), estimated using reference-based placental cell deconvolution [40], were evaluated using a linear model adjusting for gestational age, dataset, and PlaNET coordinates 2 and 3.

### Identification of site-specific sex-associated autosomal DNAme

Autosomal differentially methylated positions (DMPs) were identified in the discovery cohort by linear modeling on M values, adjusting for gestational age, dataset location of origin, and PlaNET coordinates 2 and 3. Benjamini-Hochberg (FDR) multiple test correction was performed, effect size was calculated as Δβ = Average Male β – Average Female β. In the replication cohort, a similar model was used though PlaNET-inferred ancestry was not adjusted for as this cohort was very homogeneous (predominantly European/white), see Supplementary Figure 1. DMPs were considered replicated at FDR < 0.05 and Δβ > 0.05 in the same direction as the discovery cohort.

### BLAST analysis for sex chromosome cross-hybridization

Command-line nucleotide BLAST (blastn) was performed on the 50-nucleotide probe sequences of replicated DMPs, against four versions of hg19 (in silico bisulfite converted fully methylated and fully unmethylated, both forward and reverse complement) [12]. BLAST results were considered non-specific with a match of > 40 nucleotides with > 90% sequence identity and a nucleotide match at position 50. Chen et al. and Price et al. used similar criteria [43, 44], though we chose to tolerate sequence matches with gaps in the interest of discovering even low-probability cross-reactivity to the sex chromosomes, as other studies have shown that 50-mer probes may cross-hybridize to regions with as little as 75–80% sequence identity at > 14 contiguous nucleotides [50].

### Gene ontology analyses

Gene Ontology (GO) enrichment analysis was conducted on genes associated with replicated DMPs using the “gometh” function from missMethyl, which accounts for the potential bias of multiple CpGs per gene [51]. The background set was genes associated with the 324,104 linear modeling input autosomal CpGs. Biological process GO terms satisfying FDR < 0.05 were considered significantly enriched.

### Proximity to transcription factor binding motifs

Using the CentriMo tool for local enrichment analysis from the Multiple Em for Motif Elicitation (MEME) Suite browser tool [52–54], DMPs were examined for enrichment in proximity (100-bp window with the CpG of interest at the center) to transcription factor (TF) binding motifs from the Homo sapiens Comprehensive Model Collection (HOCOMOCO) version 11 as compared to the background 324,104 autosomal CpGs. Exact goodness-of-fit-tests were used to evaluate enrichment for proximity to androgen receptor (AR) or estrogen receptor (ER) α and β binding sites, AR/ER binding site genomic coordinates were obtained from Wilson et al. and Grober et al. [55, 56].

### Relationship between sex-specific DNAme and differential gene expression

Placental gene expression data (Affymetrix Human Gene 1.0 ST Array) was downloaded for GSE75010 [57]. Non-preeclamptic samples from this cohort born > 37 weeks of gestation were analyzed (*n* = 34, 47% female). Genes within 250 kilobases of the 162 DMPs were tested for differential expression by sex, adjusting for maternal hypertension (yes/no), self-reported ethnicity, and gestational age at birth. Genes were considered differentially expressed by sex at nominal significance (*p* < 0.05).

### Further characterization of differentially methylated CpG sites

Differentially methylated genomic regions (DMRs) were defined from all 324,104 autosomal CpGs using the R package DMRcate with lamba = 1000 and C = 2 [58]. DMRs were considered significant at an FDR < 0.05 if comprised of at least 3 CpG sites with a mean Δβ (Average Male β – Average Female β) of > 0.05 in either direction. A lower Δβ was tolerated in this analysis as it was a regional average.

### Extended phenotypic analysis

Associations between clinical variables and sample scores along the first principal components (PC1) computed within each sex at the top 162 DMPs were assessed via sex-stratified linear regression in a subset of the Vancouver cohort with available extended clinical information (samples from datasets GSE74738, GSE100197, GSE109567, GSE12887, *n* = 34, 53% female). Categorical variables assessed were 450K array row, chip, and batch; positive maternal serum screen (yes/no); and delivery type (vaginal/cesarean). Continuous variables were gestational age, maternal body mass index, maternal age, birthweight, birthweight standard deviation z-score (corrected for infant sex and gestational age), processing time between delivery and placental sampling, and estimated proportions of placental cell types.

## Results

### Genome-wide measures of DNAme do not differ by placental sex

To investigate whether female (XX) and male (XY) term placentae had different global DNAme profiles, we evaluated mean genome-wide DNAme at all autosomal CpGs (*n* = 324,104) and at repetitive elements, frequently interrogated as surrogates for global DNAme as they comprise roughly 30% of all genomic nucleotides and 30% of CpG dinucleotides, specifically [59]. Mean autosomal β values did not differ by sex in this cohort (Kruskal-Wallis *p* > 0.05); sex was also not significantly associated with mean DNAme at Alu or LINE1 repetitive elements.

**Fig. 1:**
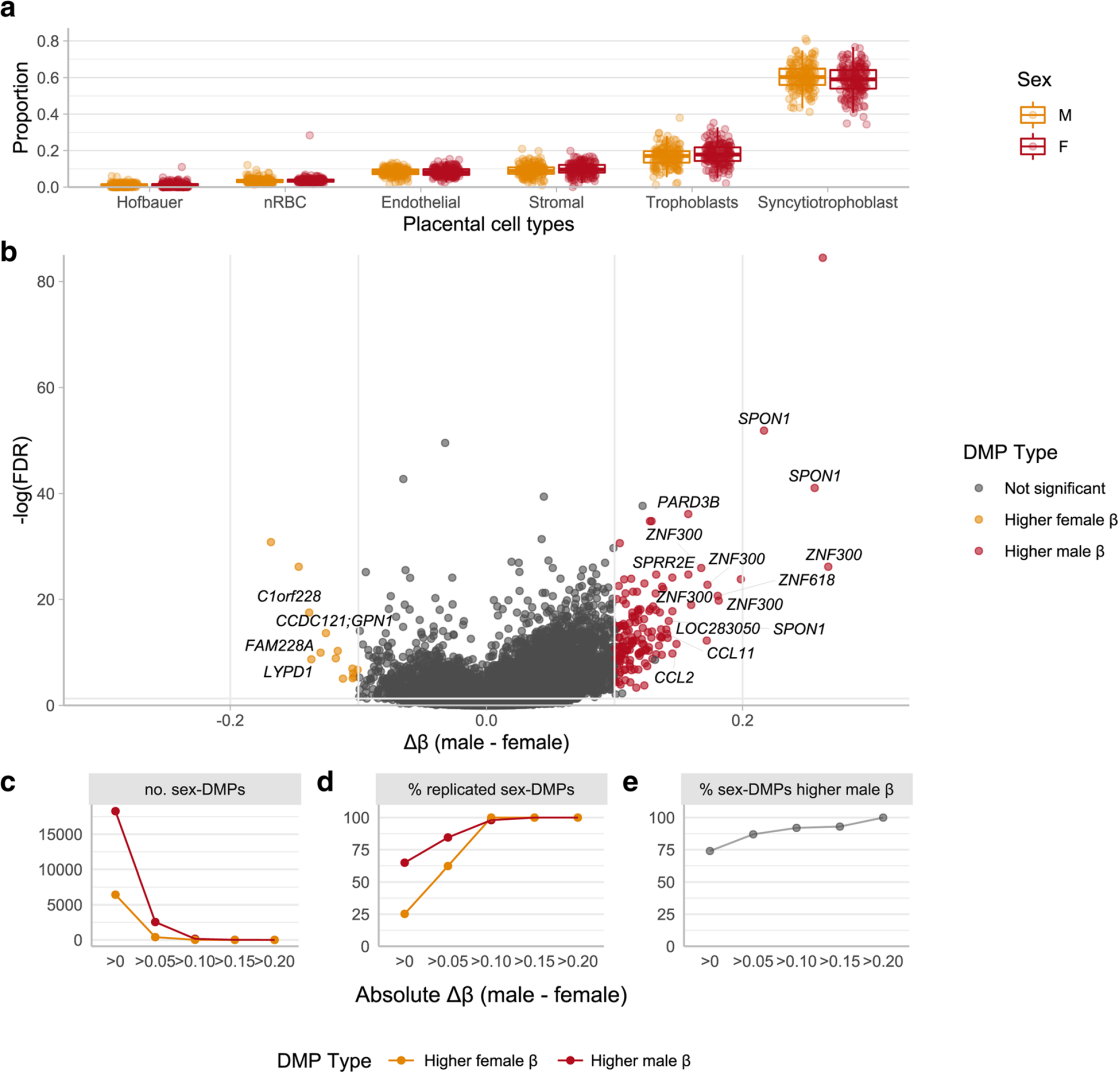
Sex differences in autosomal DNAme patterns by placental sex. **a** Cell type proportions by sex in the discovery cohort, estimated using the R package PlaNET. Cell type proportions do not significantly differ by sex (p > 0.05). **b** Volcano plot of all 324,104 autosomal CpG sites in the discovery cohort. Thresholds of statistical and biological significance are depicted by horizontal (FDR < 0.01) and vertical (∆β > 0.10) intercepts. Significantly differentially methylated autosomal CpG sites by sex (FDR < 0.01, ∆β > 0.10) are highlighted in color to indicate direction of sex-biased DNAme. CpG sites in yellow have significantly higher average male DNAme at these thresholds, and red sites exhibit higher female DNAme. CpG sites not significantly differentially methylated by sex at these thresholds are in gray. Each point represents a single CpG site, ∆β = β_avgmale_ − β_avgfemale_. The most differentially methylated CpG sites are annotated with associated genes names. **c** The number of differentially methylated (FDR < 0.05) CpG sites at various ∆β thresholds; DMPs that are more highly methylated in male samples are indicated in red, and DMPs more highly methylated in female samples are indicated in orange. **d** Percentage of DMPs at various ∆β thresholds that replicate (FDR < 0.05, ∆β same direction) in GSE71678, colored by sex with higher DNAme. **e** For all DMPs at the ∆β thresholds considered, the percentage of DMPs with higher male DNAme

When measuring DNAme in bulk tissue such as the placenta, it is important to consider how sampling procedures and/or biology may alter relative cell type proportions in a biological sample and to consider how this may be reflected in the results [60]. Considering six major placental cell types (trophoblasts, syncytiotrophoblasts, stromal cells, Hofbauer cells (placental macrophages), and endothelial cells), relative cell type proportions did not differ by sex in this cohort (Fig. 1).

### Male placentae show higher DNAme at differentially methylated autosomal CpGs

The results of linear modeling for sex-specific DNAme at various statistical (FDR) and biological (Δβ) thresholds are reported in Table 2. Significant differential DNAme was defined as FDR < 0.05 and an absolute Δβ > 0.10 between males and females; a larger effect size was chosen to focus on CpGs more likely to have biological impact, and reproduce in future studies [20]. In total, 166 sex-associated differentially methylated positions (DMPs) fit these criteria, of which 92% were more highly methylated in males than in females, a pattern observed at all thresholds considered (Fig. 1, Table 2). See Supplementary Table 1 for the results of all investigated autosomal CpGs.

**Table 2:** Linear modeling for sex-specific autosomal DNAme shows consistent higher male methylation

	Δβ > 0	Δβ > 0.05	Δβ > 0.10	Δβ > 0.20
**FDR < 0.05**	24,715 (0.74)	2,942 (0.87)	166 (0.92)	4 (1.00)
**FDR < 0.01**	14,108 (0.80)	2,682 (0.88)	166 (0.92)	4 (1.00)

Number of significantly differentially methylated autosomal positions at various statistical and biological thresholds are shown. FDR indicates the Benjamini-Hochberg false discovery rate, and Δβ refers to β value sex difference (male-female). Numbers in brackets indicate the proportion of sites at each threshold more highly methylated in male placentae

**Fig. 2:**
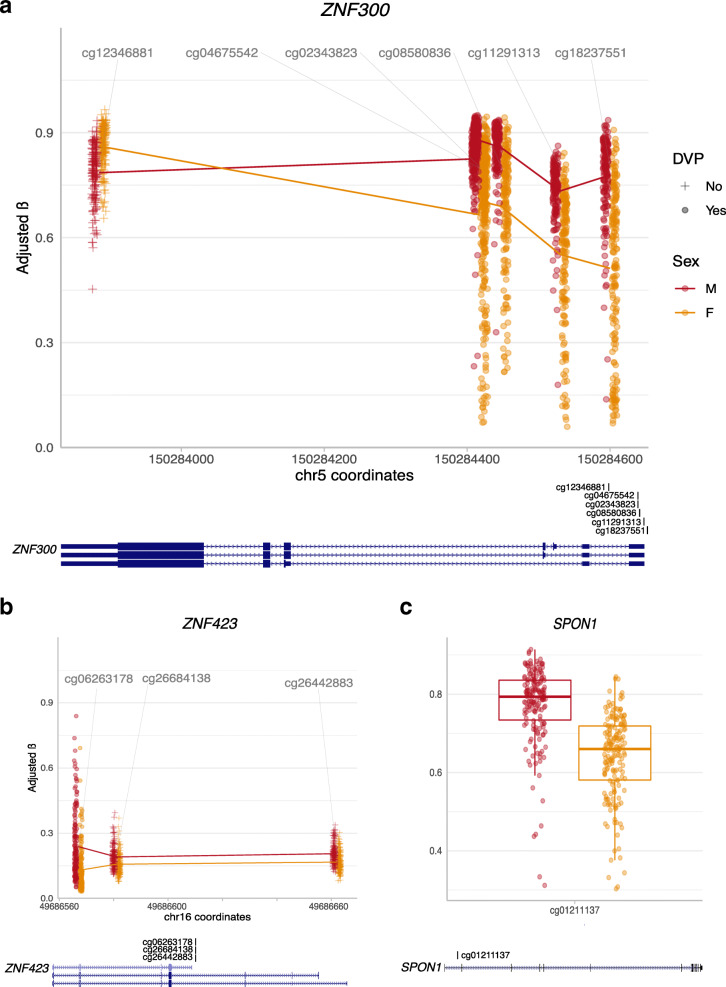
Scatterplots of sex-differentially methylated regions and probes in key genes. **a** Differentially methylated region spanning 5 CpGs in *ZNF300* in chromosome 5; male samples are indicated in red, and females in orange; the CpG coordinates along chromosome 5 are indicated on the X axis, while DNA methylation β values for each sample are plotted along the Y axis. Gene tracks from the UCSC Genome Browser with the CpG locations are indicated. **b** A differentially methylated region in *ZNF423*; coordinates along chromosome 16 are indicated on the X axis. **c** A significantly differentially methylated CpG site in the gene body of *SPON1*; this site overlaps an estrogen receptor β binding site

We hypothesized that some DMPs may comprise larger regions of correlated sex-specific DNAme, as several of the DMPs overlapped the same genes and genomic regions. DMR analysis in the discovery cohort identified 87 sex DMRs. The 87 DMRs were comprised of 435 CpGs, with an average of 5 CpGs per DMR, and ranged in size from 36 to 3306 base pairs (mean 890 base pairs); DMRs were on average 6.3% differentially methylated between the sexes. Of the 87 DMRs, 29 (33%) included one or more of the 166 identified DMPs, and conversely, 46 of the 166 DMPs (28%) were part of DMRs. It is possible that more of the DMPs are part of correlated regions of sex-biased differential DNAme, but the array coverage is not sufficient for their detection. Genes overlapping sex-specific DMRs included several from the chemokine ligand CCL family (2, 11, 13), the keratin KRT family (6, 74), the LCE family (1B, 6A), the SPRR family (1A, 2A, 2C, 4), and the ZNF family (423, 300), including *ZNF300* and *ZNF423*, see Fig. 2. *SERPINA6* overlapped a DMR more highly methylated in male samples. For a list of all identified DMRs, see Supplementary Table 2.

### Replication of sex differences in DNAme

In EWAS studies, it is important to evaluate the robustness of any findings in an independent dataset to increase the likelihood of true positive findings. For replication, linear modeling was conducted to identify DMPs by sex in an independently processed Illumina 450K dataset, GSE71678 (n = 293, 47% female). Because differences in DNAme (Δβ) are related to both biological and technical variables, and can vary for technical reasons alone by as much as 0.03–0.05, we used a less stringent Δβ threshold to define replication [20, 45]. Of the 166 DMPs identified in the discovery cohort, 98% (*n* = 163) replicated at an FDR < 0.05 and Δβ > 0 in the same direction as observed in the discovery cohort, see Fig. 1.

### Genomic cross-hybridization of probes underlying sex-specific DNAme

To exclude the possibility that the sex-specific autosomal DNAme was the result of sex chromosome cross-hybridization, we BLAST-ed the probe sequences associated with the replicated 163 DMPs against the hg19 human reference genome. Only one probe showed evidence for cross-reactivity: cg02325951, underlying a CpG in the gene body of *FOXN3.* In this probe sequence 43 nucleotides match a region on Xp, approximately 1 kb upstream of *HSD17B10* (chrX: 53467618-53467660)*.* As such, sex-specific DNAme at this CpG could not be confidently attributed to the intended genomic target (chr14: 89878619-89878668), and we elected to exclude this CpG from downstream analyses (Supplementary Figure 2). This probe has previously been reported to be differentially methylated by sex in the placenta [14].

### Characterization of autosomal sex-specific DMPs

The remaining 162 replicated and BLAST-ed DMPs were investigated for biological meaning. We observed no enrichment for being located in specific genomic regions (gene bodies, promoters, intragenic regions), on any particular autosomal chromosome, nor for their position relative to CpG islands (CpG islands, shores, or shelves). Gene ontology analysis revealed significant enrichment for 10 biological process terms, which could be largely divided into two categories, the first related to chemokines/chemotaxis and immune function (chemotaxis; eosinophil, monocyte, and lymphocyte chemotaxis; chemokine-mediated signaling; cellular response to interleukin-1), and the second related to epithelial barrier function (peptide cross-linking, keratinocyte differentiation, keratinization, and cornification).

### Association with gene expression and transcription factor binding sites

As DNAme-gene expression relationships can occur over moderate genomic distances, we tested whether genes within 250 kilobases of the 162 DMPs displayed sex-biased expression. Of these 242 genes, 11 were differentially expressed between male and female placentae (nominal p < 0.05), see Supplementary Table 3. Among the differentially expressed genes was *ZNF300*, which was more highly expressed in female samples and harbored a promoter DMP that was more highly methylated in males. *ZNF300* has been previously reported to be more highly expressed in female placentae [16].

Altered DNAme may interact with gene expression patterns by affecting the efficiency of TF binding, either positively or negatively depending on the transcription factor [61]. Binding motifs for six transcription factors were significantly overrepresented within 200 base pairs of the top DMPs (adjusted P value < 0.05 and CentriMo E-value < 1). This included motifs for AHR, ATF3, GMEB2, ZBT14, and KAISO (encoded by *ZBTB33*), see Table 3. *ZBTB33* is located on the X chromosome (Xq24), while the other transcription factors are encoded by autosomal genes. *AHR, ATF3, GMEB2, ZBTB33,* and *ZBTB14* were confirmed to be robustly expressed in the term placenta using dataset GSE75010, all five were more highly expressed than the median expression log2 counts per million of all placentally expressed transcripts.

**Table 3:** Transcription factor binding motifs overrepresented within 100bp of the top 162 DMPs

Motif ID	Coding gene	Chromosome	Consensus Seq	E-value	Adj *P* value
AHR_HUMAN.H11MO.0.B	*AHR*	7	DTYGCGTGM	0.00	5.60E− 14
ATF3_HUMAN.H11MO.0.A	*ATF3*	1	GGTSACGTGAB	0.04	5.30E− 05
GMEB2_HUMAN.H11MO.0.D	*GMEB2*	20	NBKTACGTVRN	0.00	2.50E− 08
KAISO_HUMAN.H11MO.0.A	*ZBTB33*	X	SARRYCTCGCGAGAV	0.00	9.30E− 09
KAISO_HUMAN.H11MO.1.A	*ZBTB33*	X	TMTCGCGAGAN	0.00	1.30E− 06
ZBT14_HUMAN.H11MO.0.C	*ZBTB14*	18	GGAGCGCGC	0.09	1.20E− 04

Consensus sequences are indicated with IUPAC nucleotide codes. E values refer to the central enrichment test statistic employed by CentriMo, indicating the likelihood for motif enrichment near the DMP

We further tested whether the 162 DMPs were enriched for proximity to ER α and β and AR binding sites, as molecular sex differences can arise in general from the action of either sex chromosomes or sex hormones [1]. We found no enrichment for ER α/β or AR binding sites within 200 base pairs around the CpG of interest. Only two DMPs overlapped AR and ER β binding sites, respectively; an intergenic CpG site on chromosome 8 overlapped an AR binding site, while a CpG site in the body of *SPON1* overlapped an ER β binding site, see Fig. 2.

### Limited overlap of DMPs with previous studies

To contextualize our results within the existing literature, we considered the overlap of the 162 DMPs with two previous placental DNAme studies [13, 14]. Comparisons were restricted to probes common to our discovery cohort (*n* = 324,104) and each of the previous studies’ datasets, respectively. None of the 21 autosomal DMPs reported by Martin et al. could be evaluated for overlap with our results as they were excluded for being poor quality in our discovery cohort, attributable to technical variation between the two studies [13]. However, at an FDR < 0.05, our study identified 84/335 DMPs (25%) and 154/335 DMPs in the same genes (46%) reported by Mayne et al. [14] (Table 4).

**Table 4:** Overlap of placental autosomal differentially methylated CpGs reported in this study with previous literature

Study	Martin et al. 2017	Mayne et al. 2017
Sample size (n, % female)	84 (69%)	62 (56%)
Gestational age (mean weeks)	25.5	≥ 37
Autosomal DMPs reported (n)	21	420
Autosomal DMPs with higher male β (%)	62%	100%
Autosomal DMP probes covered in this study (n)*	0/21	335/420
Overlap with present study		
FDR < 0.05, Δβ > 0.10 (*n* = 162)	-	0/335
FDR < 0.05, no Δβ (*n* = 24,715)	-	84/335
Genes at FDR < 0.05 (*n* = 6,733)	-	154/335

*Due to differences in probe filtering, not all DMPs reported in previous studies were in the filtered dataset of 324,104 autosomal CpGs used here, overlap only considered for common CpGs

### Combined effect of sex-specific DNAme at DMPs

**Fig. 3:**
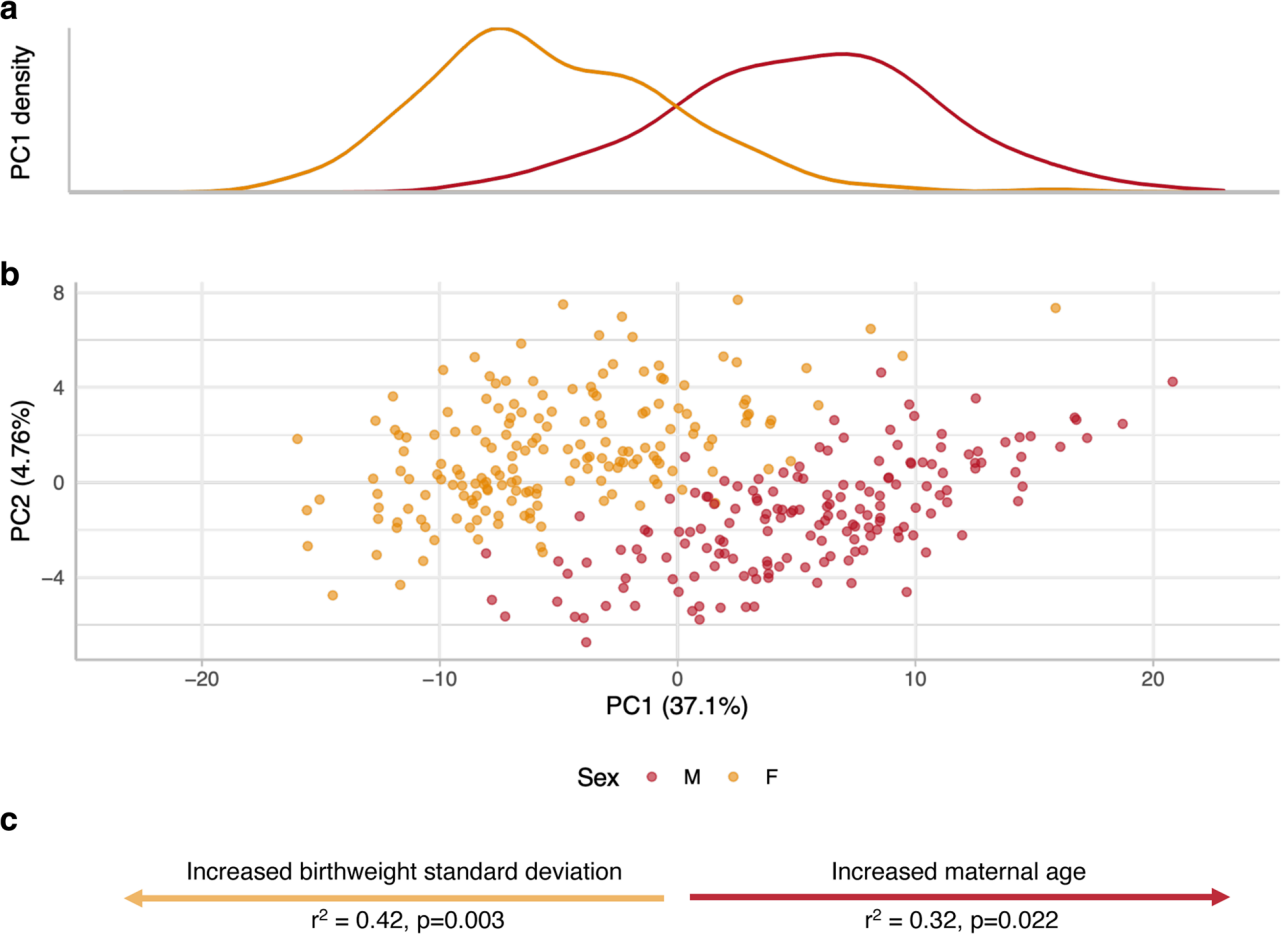
Principal components analysis of DNAme at the 162 significantly sex-differentially methylated CpGs. **a** Density plot of male (M, red curve) and female (F, yellow curve) samples along PC1 of the 162 sex-associated DMPs. **b** Scatterplot of PC1 versus PC2 of the discovery cohort; male (M) samples are plotted in red, and females (F) in yellow. **c** Significant associations between clinical variables and PC1 in a sex-stratified analyses. Linear regression R^2^ and p values are reported for each significant variable; a yellow arrow indicates significant association in females, and a red arrow indicates significant association in males. PC indicates principal component

To evaluate the cumulative effects of sex-specific DMP methylation, we performed principal components analysis on the β values associated with these 162 CpGs in all samples. PC1 (37.1% variance) and PC2 (4.76% variance) were significantly associated with sample sex (ANOVA *p* < 0.05, respectively), and male and female samples formed overlapping clusters along PC1 (Fig. 3).

As sex biases are observed in the frequency and severity of many pregnancy complications, we hypothesized that PC1 may be associated with sex-specific clinical features, such as infant birthweight. When considering both sexes together, no clinical or technical characteristics were significantly associated with sample position along PC1, although PC1 only explains a portion of the variance by sex at these loci (37.1%) so this is not unexpected. However, when sex-stratifying PCA and association analyses, maternal age was significantly positively associated with PC1 in males, while birthweight standard deviation was significantly positively associated with PC1 in females.

We also leveraged PCA to investigate the relationship between DMP methylation and gestational age and sex chromosome complement. Twenty-four second and early third trimester samples (21–32 weeks), including three with 45,X chromosome complements, were projected into the PCA space associated with the 162 DMPs in the discovery cohort. Second and early third trimester male and female samples localized to the top half of the plot, indicating that PC2 is associated with gestational age (*p* < 2.2e− 16). The 45,X samples were found to localize to the male cluster along PC1 within the younger gestational age samples, suggesting a possible relationship between X chromosome complement and DNAme at these DMPs, see Supplementary Figure 4.

## Discussion

In this study on human placenta, we identified 162 DMPs across all autosomes that showed robust DNAme differences by placental sex. Of the 162 sex-associated DMPs, over 90% were more highly methylated in male placentae, confirming a previous observation [13]. Interestingly, most somatic tissues display the opposite pattern; the majority of sex-associated DMPs are more highly methylated in female samples [62] in blood [63, 64], buccal swab [63], prefrontal cortex [65], pancreatic islets [66], and also in a meta-analysis of 36 somatic tissues [62]. Additionally, a study of placental DNAme by whole-genome oxidative bisulfite sequencing identified that male placentae are on the order of 1–2% more highly methylated overall than females [67]. Though we saw no significant difference in array-wide mean DNAme by sex, this could be related to the uneven probe distribution of the 450K array, with coverage concentrated in functionally relevant areas [68].

While the underlying cause of higher DMP DNAme in males in unclear, our investigation into a limited number of placentae with a 45,X karyotype may suggest a role for X chromosome dosage. Studies of sex chromosome aneuploidies have revealed extensive influences of X chromosome dosage on DNAme profiles autosomal loci, for example in females affected by Turner syndrome (45,X) and males affected by Klinefelter syndrome (47,XXY) [69, 70]. Additionally, it has been proposed that X-chromosome inactivation may be less complete in the human placenta as compared to somatic tissues [71], and it is possible that the placental inactive X interacts differently with autosomal loci than in somatic tissues. A further link between DMP DNAme profiles and the X chromosome was found in the enrichment for overlap with KAISO protein binding motifs. KAISO is a transcription factor encoded by the X-linked *ZBTB33* gene, and has been reported to repress gene expression by binding methylated DNA [72]. *ZBTB33* being X-linked may imply the existence of interactions between sex chromosomal and autosomal loci in the placenta. Furthermore, we found no association of DMPs with nearby ER or AR binding sites, making it less likely that hormone effects underly these differences.

Genes overlapping the top 162 DMPs were enriched for biological process gene ontology terms related to chemokines and chemotaxis, as well as to the process of keratinization. This may suggest that the placenta mediates sex differential immune function and/or placental trophoblast structure or function during gestation, as genes from the KRT or keratin gene family are often used as cell-surface markers of placental trophoblasts [73], the most abundant placental cell type [74]. Several genes from the ZNF family also overlapped DMPs and DMRs. *ZNF423* and *ZNF300*, specifically, overlap DMPs that are more highly methylated in males, and are DNA-binding Krüppel-like C2H2 zinc finger transcription factors [75]. *ZNF300* has been reported to be more highly expressed in female placentae in a study of first trimester conceptuses [16], this is consistent with the higher male DNAme in the *ZNF300* promoter we observe here (Fig. 2). *ZNF423* was recently reported to regulate networks of gene co-expression (co-expression modules) in the human placenta that are conserved across gestation [15]. Along with the *ENF1* gene, *ZNF423* regulated the most highly conserved placental co-expression module between humans and mice, suggesting the importance of *ZNF423* in the regulation of patterns of placental gene expression. To our knowledge, sex differences in placental DNAme of *ZNF423* have not previously been reported, nor were sex differences in the *ZNF423* co-expression module reported. The sex-specific DNAme observed in this study across *ZNF423* could suggest that the conserved placental co-expression module identified by Buckberry et al. may be regulated in a sex-specific manner. For the plots shown in Fig. 2, the location of all CpG sites shown aligned with the RefGene and ChromHMM tracks from the UCSC Human Genome Browser [76] are available in Supplementary Figure 3.

To understand the extent to which our DMPs were related to sex differences in placental gene expression, we investigated placental microarray expression data for genes within 250 kilobases of the 162 DMPs. We observed higher female expression of *ZNF300*, consistent with previous results as discussed above. However, though 4% of these 242 genes showed sex-specific expression, the majority (96%) were not significantly differentially expressed in the placenta by sex. This may be related to the small sample size of the gene expression cohort utilized (*n* = 34), the role of additional factors beyond DNAme in regulating gene expression, and the possibility of alternative splicing and sex-specific isoform expression, which would not be captured in microarray analysis [77].

Principal components analysis found that PC1 was associated with increased maternal age in male samples, and increased birthweight standard deviations in female samples. While maternal age has been positively associated with increased risk of preeclampsia development, we are not aware of sex differences in preeclampsia risk by maternal age [78]. Conversely, birthweight standard deviation is a metric that is calculated using sex- and gestational age-adjusted growth curves [79], and as such is independent of both sex and gestational age. Although birthweight standard deviation did not differ significantly by sex, within female samples, a higher birthweight standard deviation was associated with those samples localizing toward the female extreme of PC1. To our knowledge, this is the first report suggesting that placental molecular features may interact with within-sex birthweight distributions.

In comparing the DMPs discovered in this study to findings previously reported in the human placenta [13, 14] we observed limited overlap, although all of the 85 DMPs from our study overlapped with previous reports were differentially methylated in the same direction by sex as previously reported. Limited overlap may partially relate to cohort size, as the cohort used in this study is larger than any used previously (341 samples versus 62 and 84 samples), increasing our power to detect true positive sex differences. Despite imperfect overlap with previous studies, we observed a high degree of DMP reproducibility between our discovery and replication cohorts, suggesting that the 162 DMPs identified here show consistent sex differences in placental autosomal DNA.

We acknowledge several limitations of our findings. First, the discovery cohort samples are inferred to be largely of European and East Asian ancestry, and the replication dataset is comprised exclusively of European ancestry samples [26], as such our results may not generalize to other ancestral populations. This is a limitation applying to nearly every large-scale epigenome or genome-wide association study [80, 81], and inclusion of samples of diverse ancestry should be considered in the construction of future cohorts. Second, the Illumina 450K array does not provide coverage of all genomic CpGs, specifically in non-coding regions, and future investigations using higher-resolution technologies such as whole-genome bisulfite sequencing would be valuable. We also acknowledge that by term, both sex chromosome complement and sex hormone levels have had ample opportunity to exert their effects, and thus we cannot disentangle which patterns of sex-specific DNAme observed may be related to each.

## Perspectives and significance

In summary, we find that autosomal sex differences in DNAme exist in the human placenta, and in contrast to somatic tissues the majority of placental autosomal sex-differentially methylated CpG sites are more highly methylated in male samples. These results are intended to establish a baseline for DNAme sex differences existing in the uncomplicated term placenta, and we anticipate that they will be useful to contextualize results of analyses from the placentae associated with sex-specific pregnancy complications such as preterm birth and early-onset preeclampsia.

## Supplementary Information

**Additional file 1: Supplementary Figures.** Title: Supplementary figure files. Description: Supplementary figures 1-4 with corresponding titles and figure captions.

**Additional file 2: Supplementary Table 1.** Title: Results of linear modelling for all 324,104 autosomal CpGs tested. Description: Linear modelling statistics for sex differential methylation analysis at all 324,104 autosomal CpG sites in the filtered dataset.

**Additional file 3: Supplementary Table 2.** Title: Table of significant placental autosomal sex-associated DMRs. Description: Summary statistics and genomic locations of all signficant sex-associated DMRs identified.

**Additional file 4: Supplementary Table 3.** Title: Differential expression analysis of genes within 250 kilobases of the 162 DMPs. Description: Linear modelling statistics for sex differential gene expression analysis at the 242 genes within 250 kilobases of the 162 autosomal sex-differentially methylated positions.

## Data Availability

All datasets used are publicly available via the Gene Expression Omnibus at the indicated accession numbers (https://www.ncbi.nlm.nih.gov/geo/).

## Abbreviations

450K: Illumina HumanMethylation450 Array
ANOVA: Analysis of variance
AR: Androgen receptor
BLAST: Basic local alignment search tool
BMIQ: Beta-mixture quantile normalization
CpG: Cytosine-guanine dinucleotide
DMP: Differentially methylated position (1 CpG)
DMR: Differentially methylated genomic region (> 1 CpG)
DNAme: DNA methylation
ER: Estrogen receptor
FDR: Benjamini-Hochberg false discovery rate
LGA: Large birthweight for gestational age
PC: Principal component
PCA: Principal components analysis
PlaNET: Placental DNAme Elastic Net Ethnicity Tool
SD: Standard deviation
SGA: Small birthweight for gestational age
XCI: X-chromosome inactivation
